# Supplementary material for: Cryo-EM structure of the botulinum neurotoxin A/SV2B complex and its implications for translocation
Source: Nat Commun. 2025 Feb 11;16:1224. doi: 10.1038/s41467-025-56304-z (PMC11814414; doi:10.1038/s41467-025-56304-z)
Supplement: Supplementary file 1 — Supplementary Information [file 41467_2025_56304_MOESM1_ESM.pdf]

Supplementary Information

Cryo-EM structure of the botulinum neurotoxin A/SV2B complex and its implications for translocation

Basavraj Khanppnavar[1#], Oneda Leka[1#], Sushant K. Pal[1], Volodymyr M. Korkhov[1,2*] and Richard A. Kammerer[1*]

[1] PSI Center for Life Sciences, CH-5232 Villigen, Switzerland.[2] Institute of Molecular Biology and Biophysics, ETH Zurich, Zurich, Switzerland.

[#] These authors contributed equally to the study

[*] Corresponding Authors:
Richard A. Kammerer, PSI Center for Life Sciences, CH-5232 Villigen PSI, Switzerland. E-mail: richard.kammerer@psi.ch
Volodymyr M. Korkhov, PSI Center for Life Sciences, CH-5232 Villigen PSI, Switzerland, and Institute of Molecular Biology and Biophysics, ETH Zurich, Zurich, Switzerland. E-mail: volodymyr.korkhov@psi.ch

Supplementary Figures and Tables

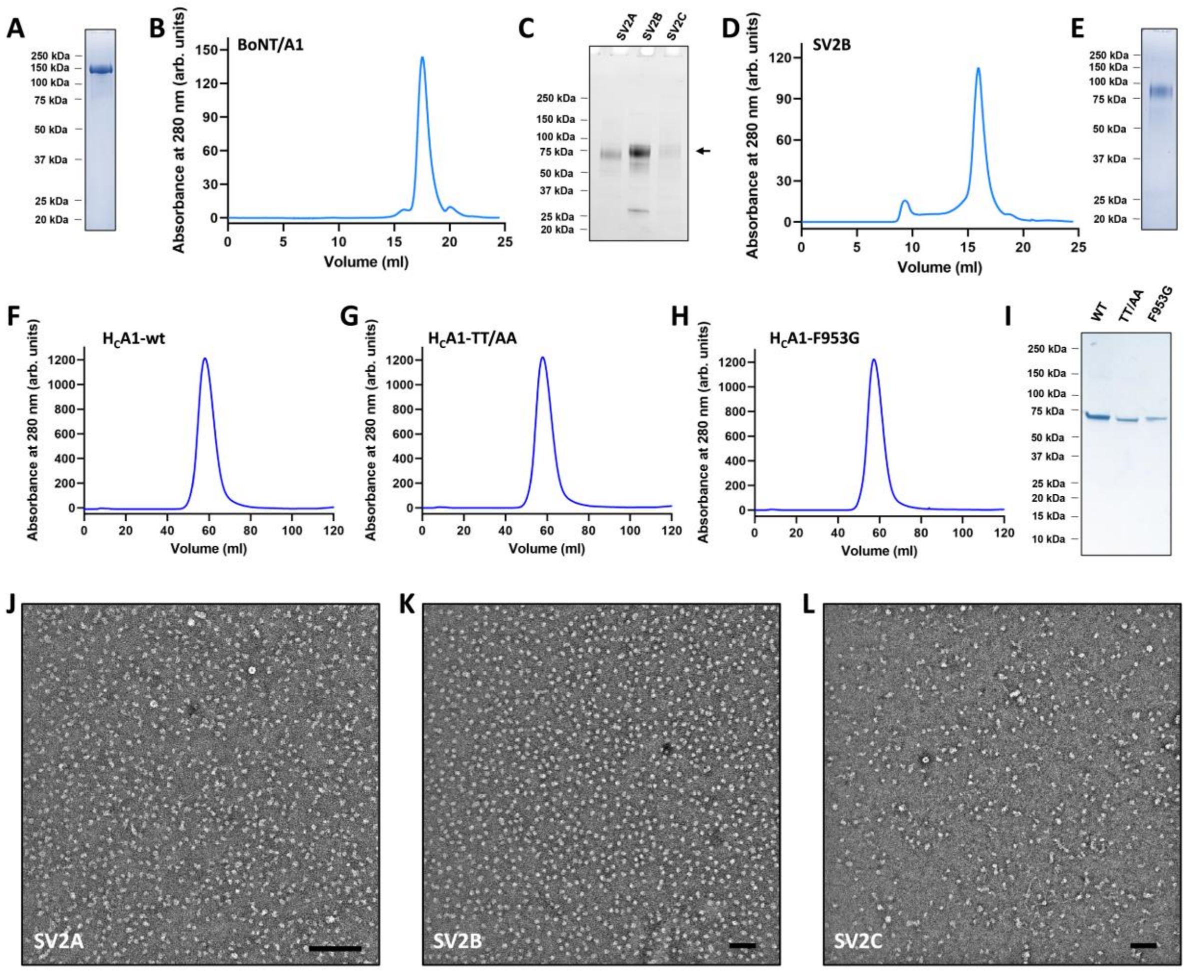

Supplementary Fig. 1. Expression, purification, and characterization of BoNT/A1 and SV2 proteins. **A, B** SDS-PAGE and size-exclusion chromatography (SEC) profile of purified inactive BoNT/A1. **C** SDS-PAGE analysis of SV2A, SV2B and SV2C purified on a small-scale using a Strep-affinity tag. **D, E** SEC and SDS-PAGE profile of SV2B after large-scale purification. **F-H** SEC profile of GST-tagged H_CA1-wild type (panel F), H_CA1-TT/AA mutant (panel G), and H_CA1-F953G mutant (panel H). **I** SDS-PAGE profile of purified wild-type and mutant H_CA1 proteins. SEC of SV2B and BoNT/A1 was performed on a Superose 6 10/300 Increase column and SEC of H_CA1 variants of BoNT/A1 with a HiLoad 16/60 Superdex 200 column. **J-L** Negative staining EM of purified SV2A (panel J), SV2B (panel K) and SV2C (panel L). Scale bar, 50 nm.

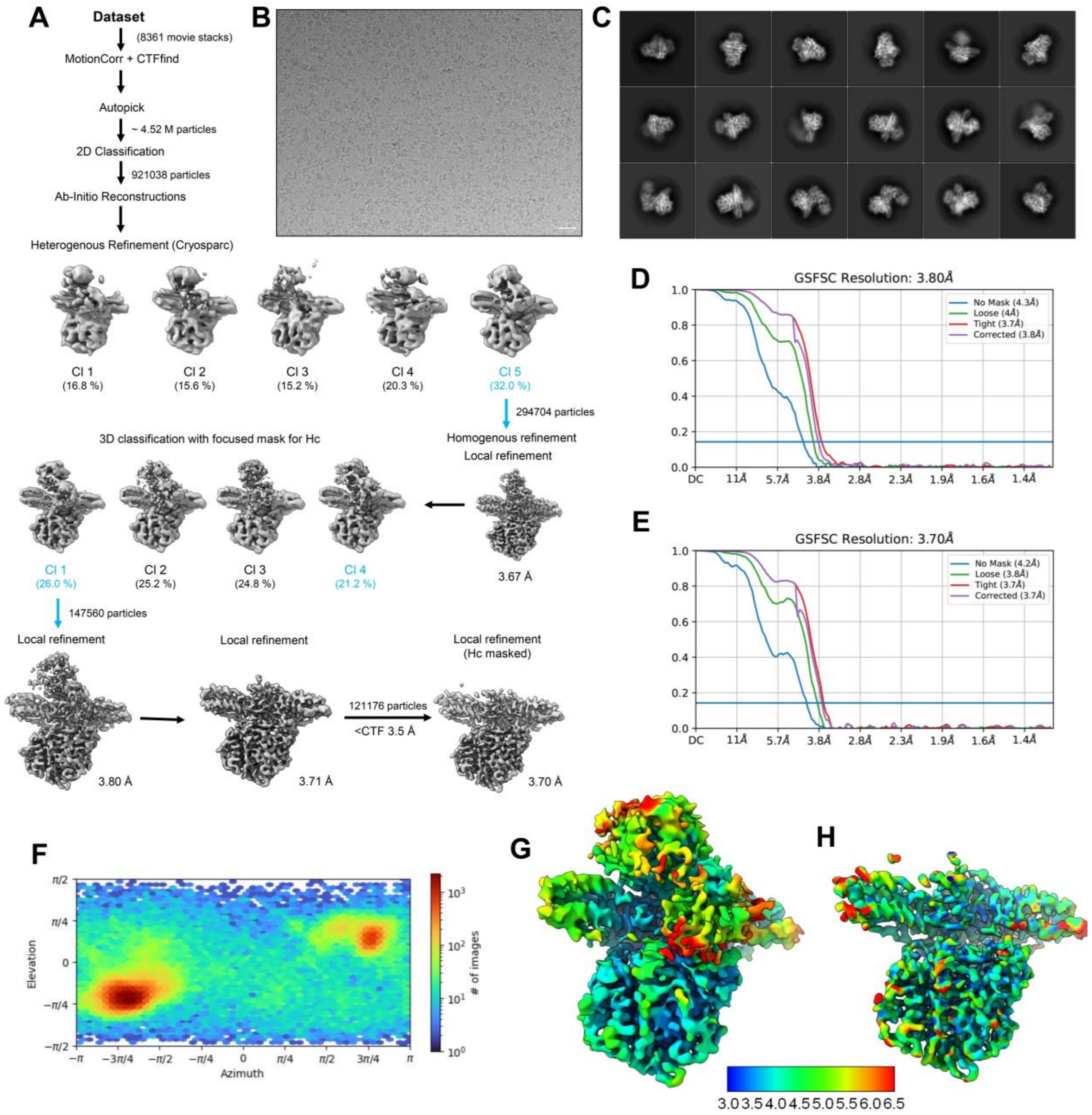

Supplementary Fig. 2. Cryo-EM data processing of BoNT/A1. **A** Schematic representation of the cryo-EM data processing pipeline. **B** Representative micrographs. Scale bar, 20 nm. **C** Representative 2D class averages. **D** Gold standard Fourier Shell Correlation curve (GFSC) curve at 0.143 cut-off for the BoNT/A1 3D reconstruction. **E** GFSC curve for the LCH_NA1 fragment of BoNT/A1 after focused refinement. **F** Angular plot of particles used for 3D reconstruction. **G, H** Local resolution map of BoNT/A1 and the LCH_NA1 fragment after focused refinement.

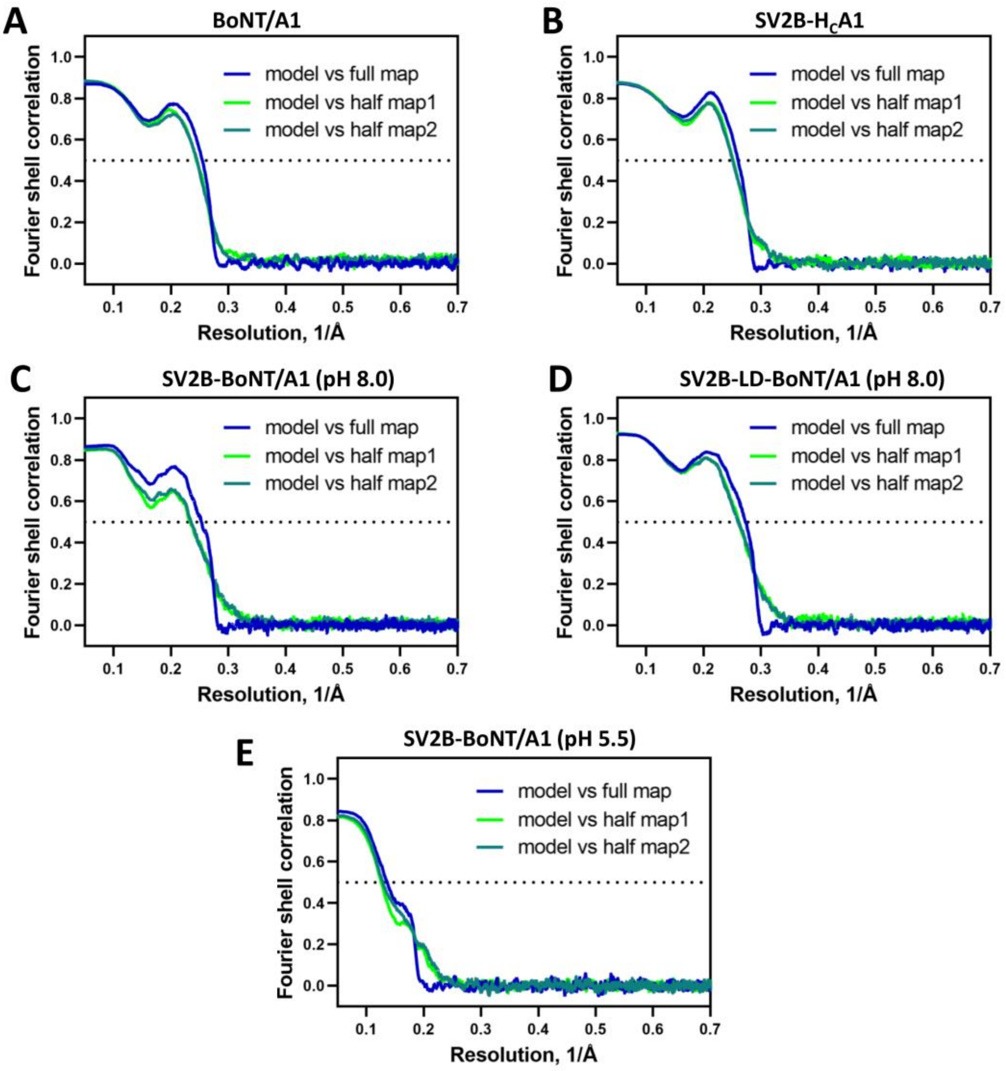

Supplementary Fig. 3. Map to model Fourier shell correlation (FSC) plots. **A, B** Map to model FSC plots of BoNT/A1 (panel A) and SV2B-H$_C$A1 (panel B) based on the final refined models. **C, D** Map to model FSC plots for the full-length SV2B-BoNT/A1 complex at pH 8.0 (panel C) and the SV2B-LD-BoNT/A1 region (panel D) after focused refinement. **E** Map to model FSC plot of SV2B-BoNT/A1 complex at pH 5.5.

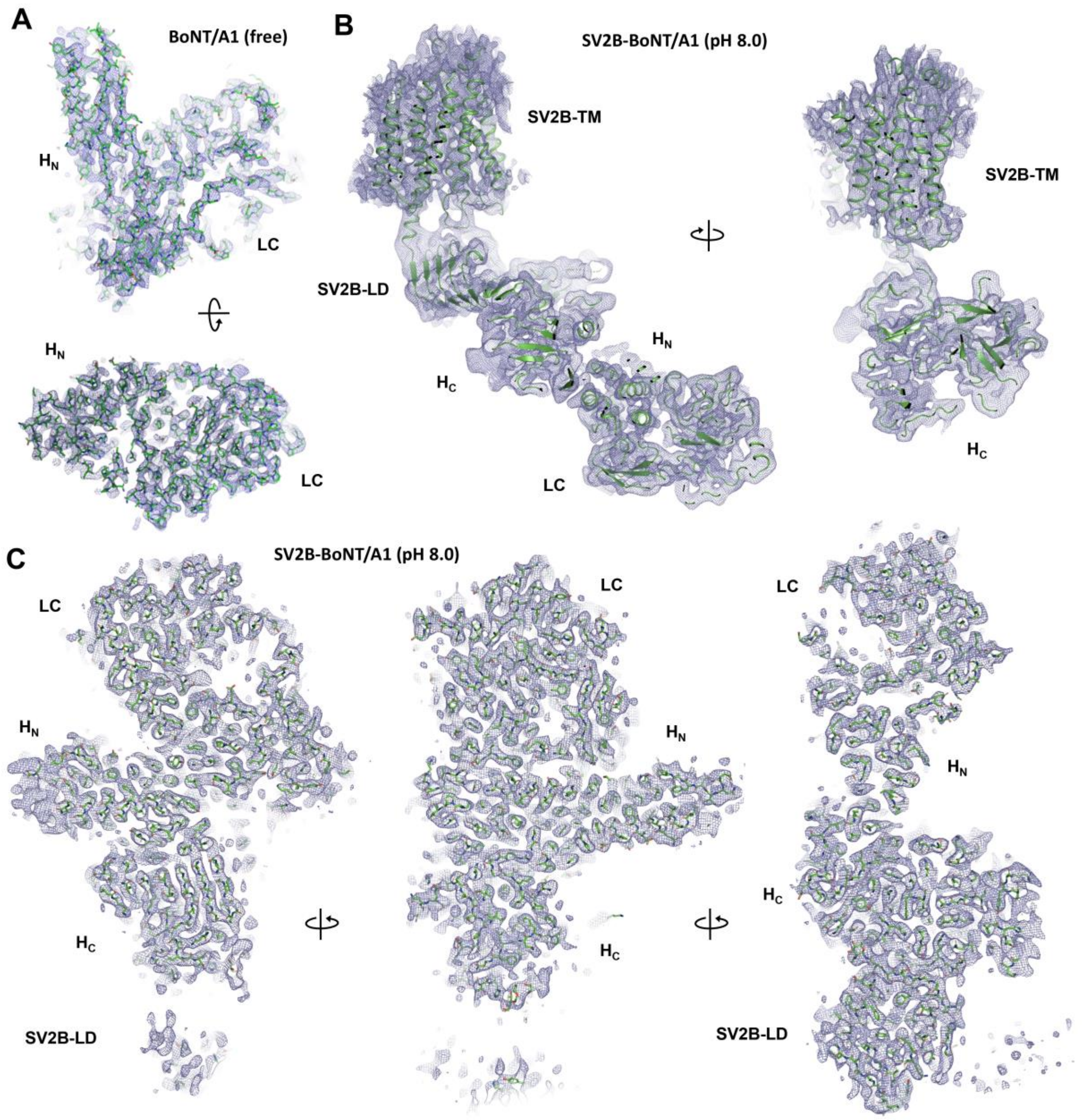

Supplementary Fig. 4. Cryo-EM map features of the free and SV2B-bound BoNT/A1 at pH 8.0. **A** Density maps for LC and H_N domains of free BoNT/A1 after focused refinement. **B, C** Density features of the SV2B-BoNT/A1 complex at pH 8.0. **C** Density features of the SV2B-BoNT/A1 complex at pH 8.0 after focused refinement. All the maps are contoured at 8σ threshold level.

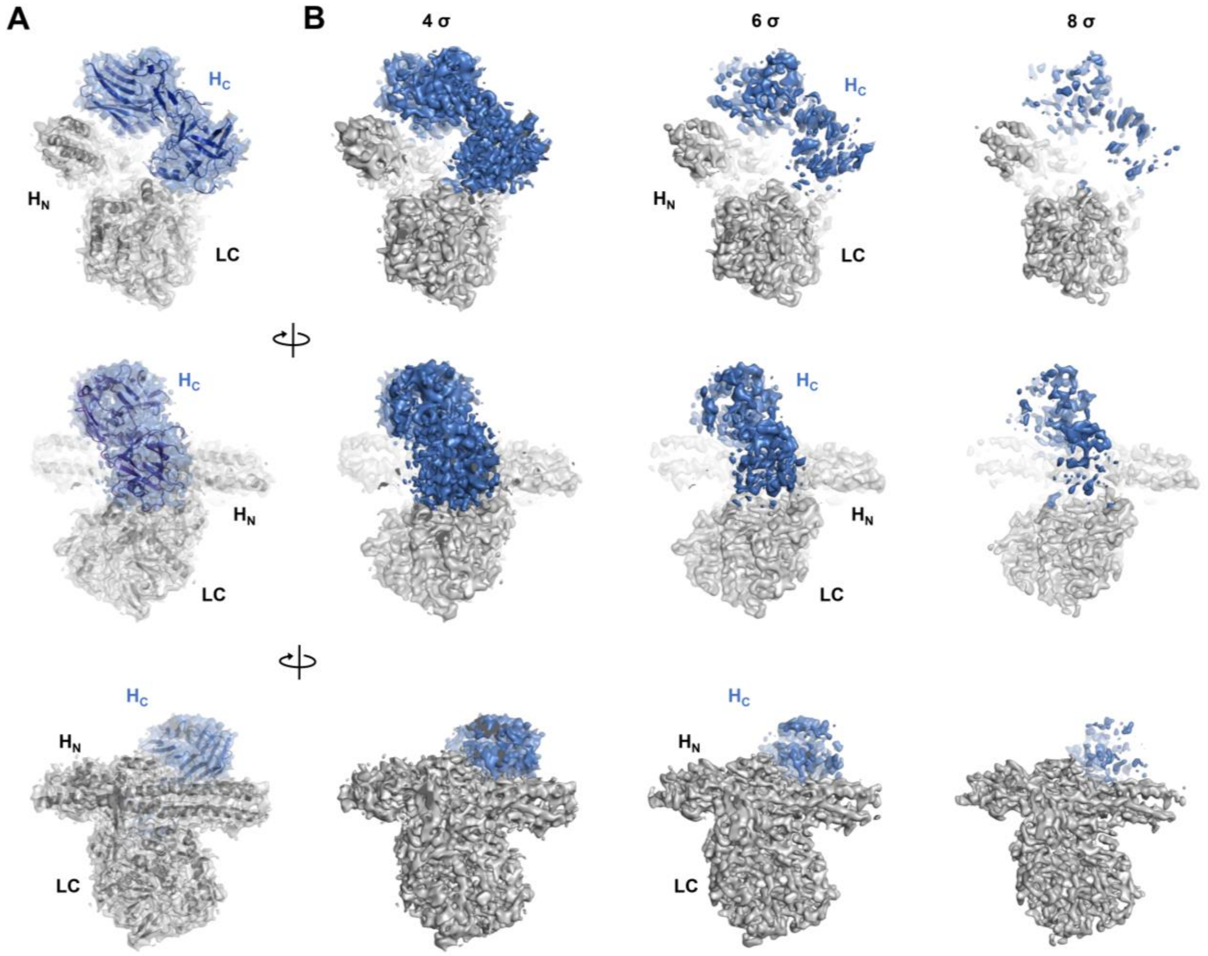

Supplementary Fig. 5. Cryo-EM map features of BoNT/A1. **A** Overlay of hybrid model BoNT/A1 consisting of the cryo-EM model and the AlphaFold model on the cryo-EM map. **B** Electron density features of the LCH_N (grey) and H_C fragments of BoNT/A1 contoured at 4σ, 6σ and 8σ threshold level.

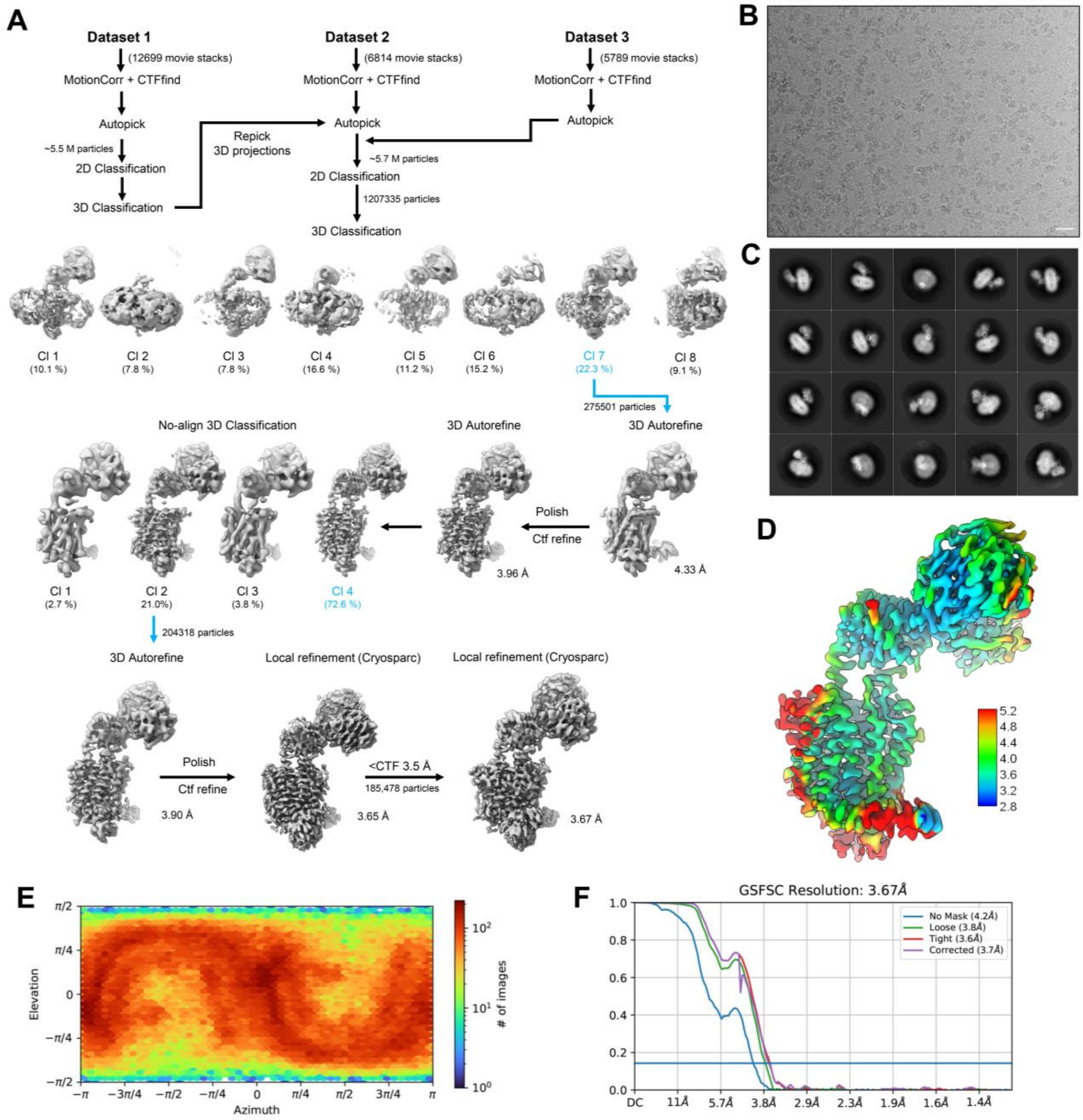

Supplementary Fig. 6. Cryo-EM data processing of the SV2B-H_CA1 complex. **A** Schematic representation of the cryo-EM data processing pipeline. **B** Representative micrographs. Scale bar, 20 nm. **C** Representative 2D class averages. **D** Local resolution map of SV2B-H_CA1. **E** Angular plot of particles used for final 3D reconstruction. **F** GFSC curve for SV2B-H_CA1.

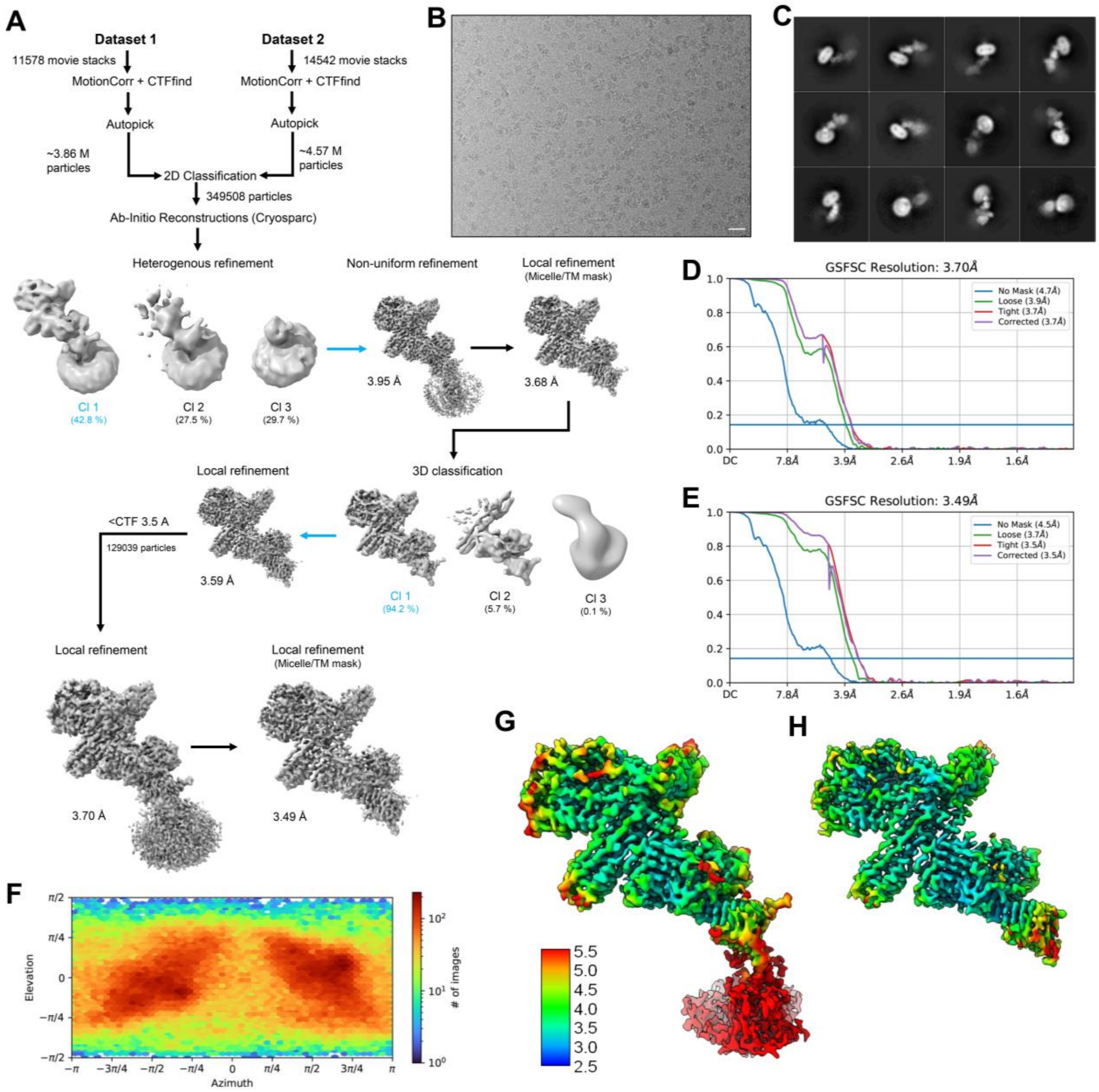

Supplementary Fig. 7. Cryo-EM data processing of the SV2B-BoNT/A1 complex at pH 8.0. **A** Schematic representation of the cryo-EM data processing pipeline. **B** Representative micrographs. Scale bar, 20 nm. **C** Representative 2D class averages. **D, E** GFSC curve for overall map of SV2B-BoNT/A1 and SV2B-LD-BoNT/A1 after focused refinement. **F** Angular plot of particles used for 3D reconstruction. **G, H** Local resolution map of SV2B-BoNT/A1 and the SV2B-LD-BoNT/A1 complex after focused refinement.

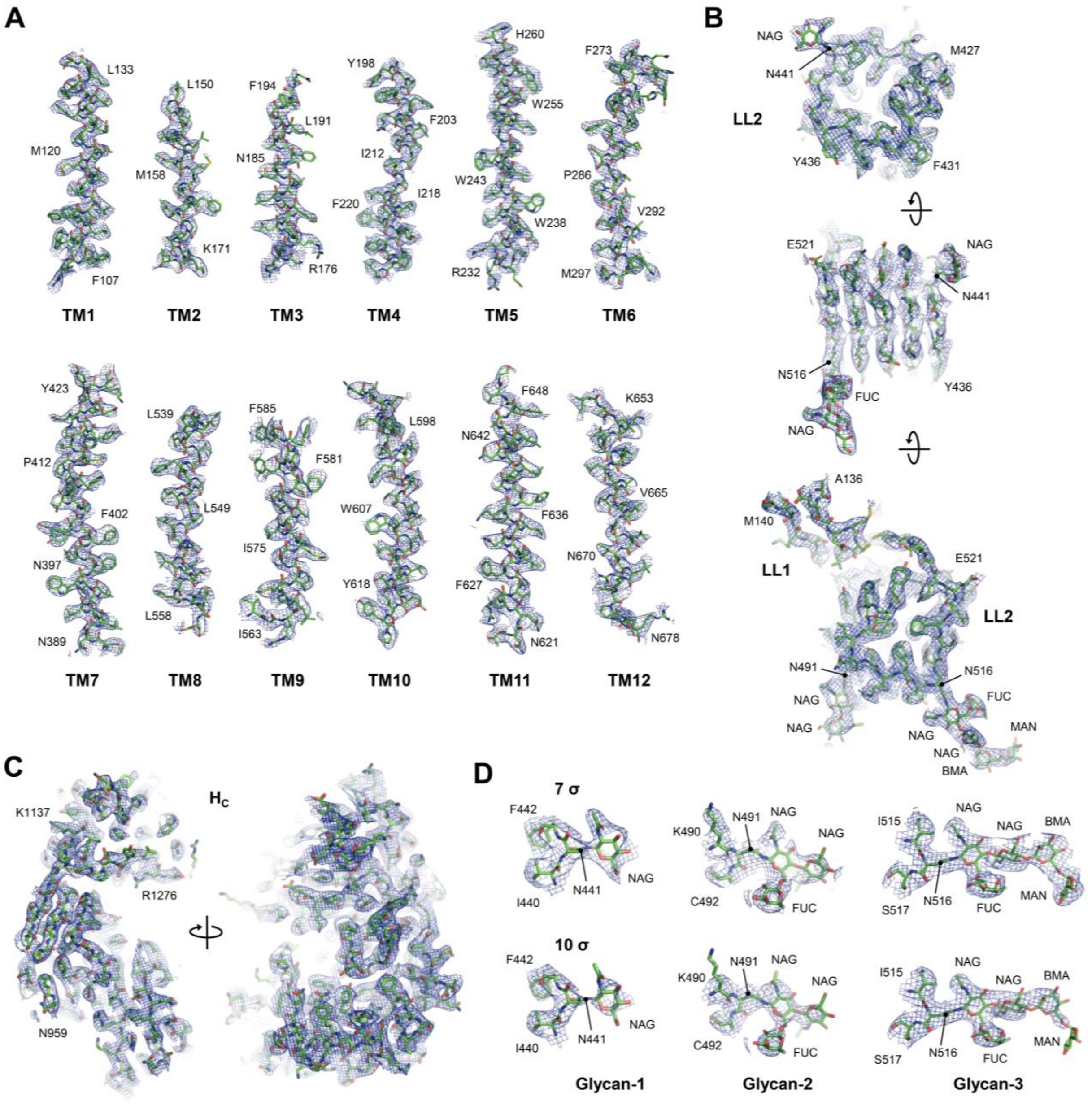

Supplementary Fig. 8. Cryo-EM map features of the SV2B-H_CA1 complex. **A** Isolated density maps for the 12 TM helices of SV2B contoured at 10σ threshold level. **B** Density features of SV2B luminal loops and **C** H_CA1 contoured at 12σ levels. **D** Density features of N-linked glycans of SV2B contoured at 7σ and 10σ threshold level.

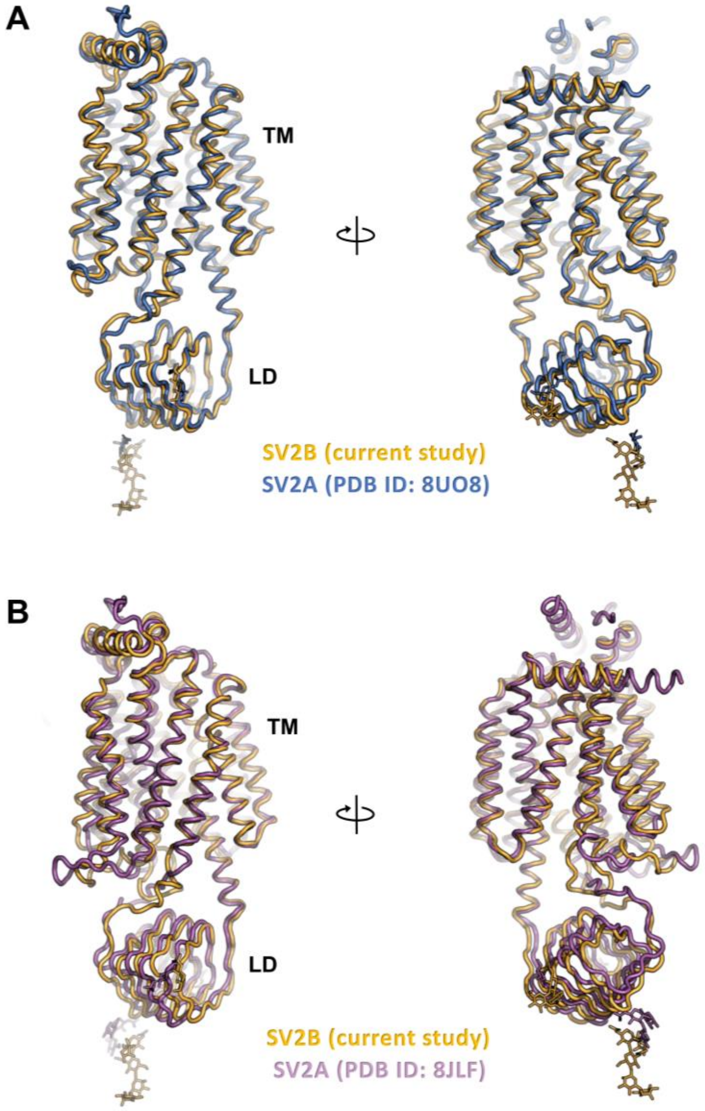

Supplementary Fig. 9. Comparison of SV2A and SV2B cryo-EM structures. **A, B** Overlay of our SV2B structure (orange) with recently the determined structures of SV2B (blue, PDB8UO8) and SV2A (magenta, PDB8JLF). The root mean square deviation (RMSD) based on an all-atom alignment of our SV2B structure with the recently reported structures of SV2B and SV2A is 0.93 Å and 1.81 Å, respectively.

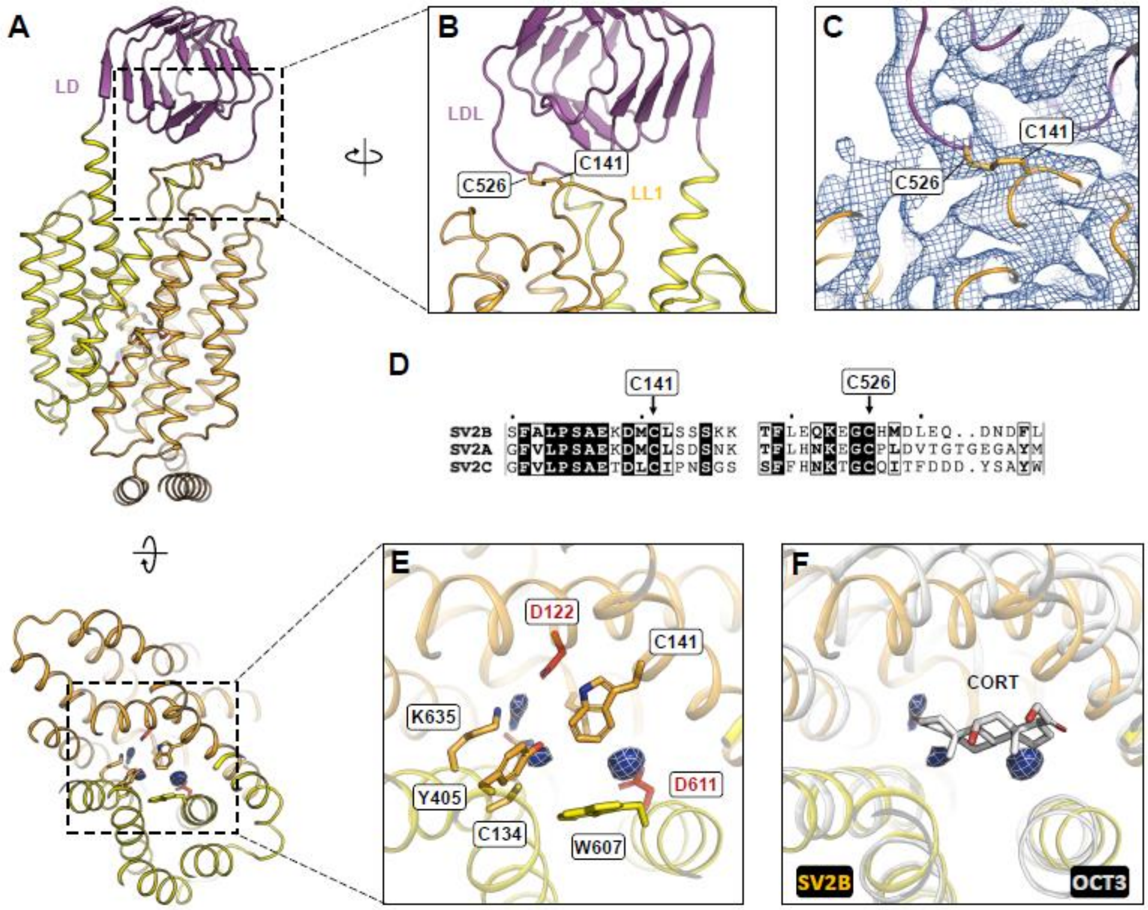

Supplementary Fig. 10. Key structural features of SV2B. **A** Overview of the SV2B structure. **B** Depiction of the disulfide bond between C141 in LL1 and C526 in LD. **C** Electron density features of disulfide bond contoured at 5σ threshold levels. **D** Multiple sequence alignment showing conservation of the two Cys residues in SV2 isoforms. **E** Density features contoured at 8σ in close proximity of the negatively-charged residues in the putative substrate-translocation pathway of SV2B. **F** Comparison of SV2B and OCT3 (PDB7ZH6) substrate binding sites revealing unidentified density of SV2B in a similar region as that occupied by corticosterone (CORT) in OCT3.

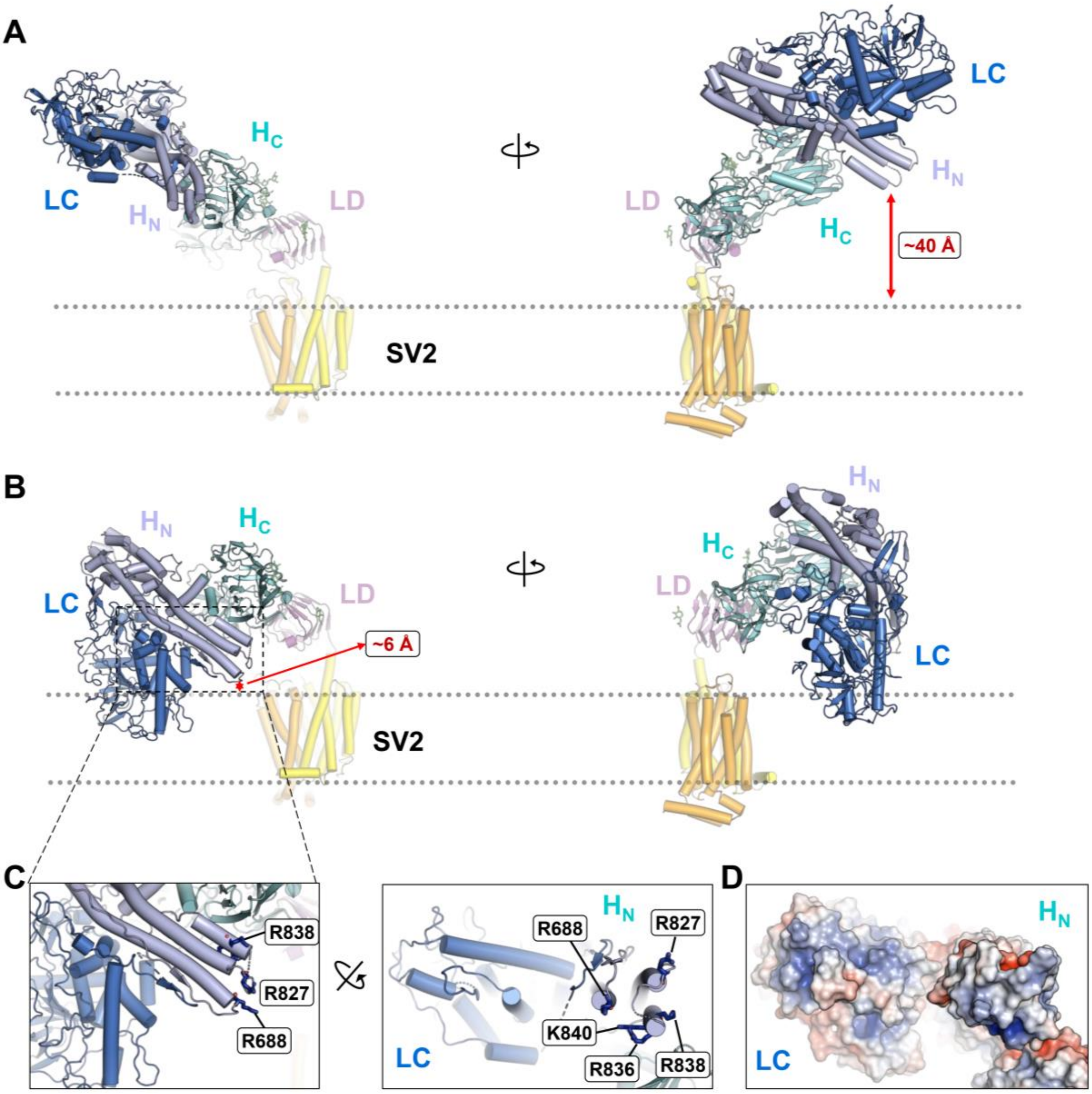

Supplementary Fig. 11. Comparison of the SV2B-BoNT/A1 complex at physiological and low pH. **A** At higher pH, the H_N and the LC domains of BoNT/A1 bound to SV2B are positioned far away from the plasma membrane or the synaptic vesicle membrane, with distances of ~40 Å and ~60 Å, respectively, from the lipid bilayer margin. **B** At acidic pH conditions (pH 5.5), the conformational changes in BoNT/A1 bound to SV2B position the LC and the H_N in translocation-competent state with distances close to and ~6 Å, respectively, to the vesicle membrane lipid bilayer margin. **C, D** Close-up views of the H_N and the LC domains showing key basic residues in the proximity of lipid bilayer (C) H_N and LC domains in surface representation colored according to electrostatic potential (scale, -5 kT/e red, +5 kT/e blue) calculated with the Adaptive Poisson-Boltzmann Solver (APBS) module in Pymol (D). Views in C (right panel) and D are identical.

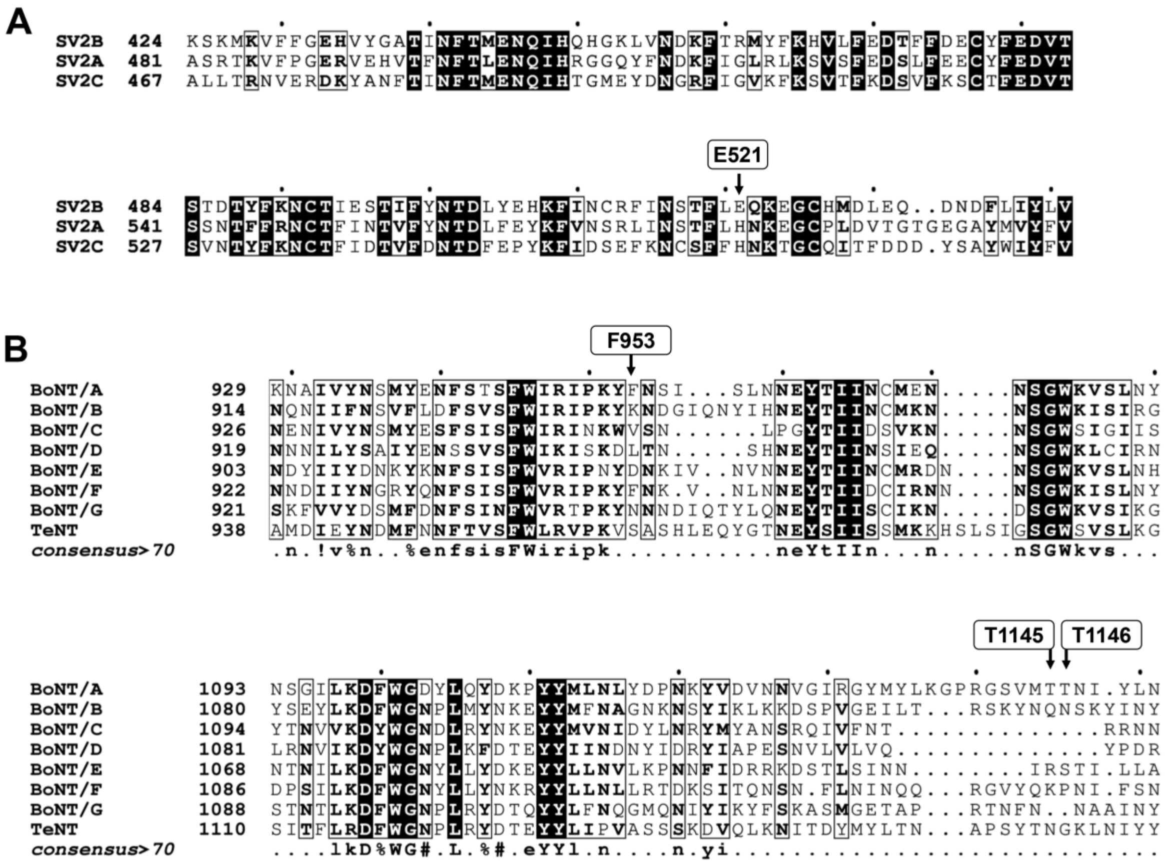

Supplementary Fig. 12. Multiple sequence alignments of SV2 proteins and BoNTs. Residues that are critical for the interaction are indicated.

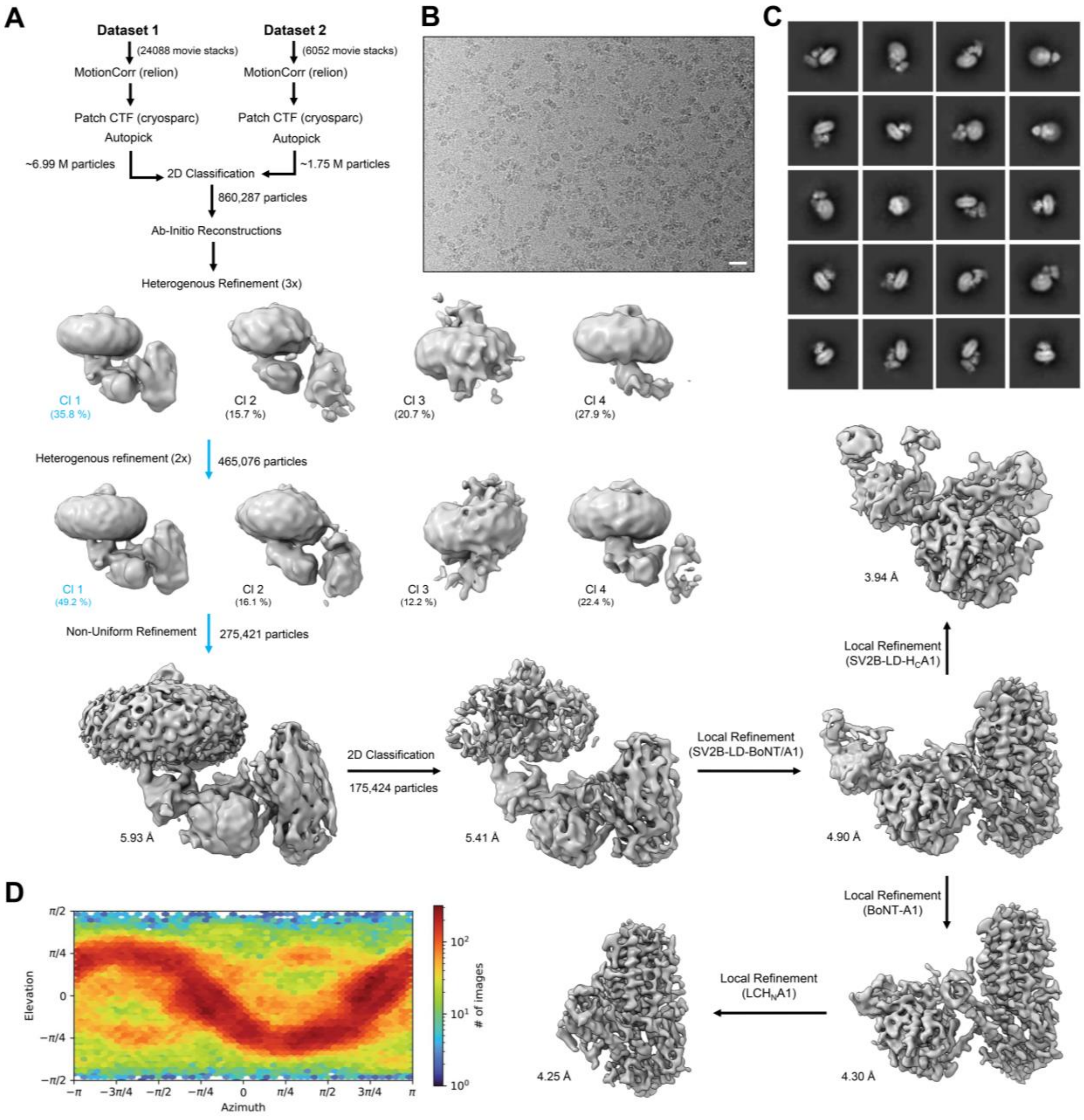

Supplementary Fig. 13. Cryo-EM data processing of the SV2B-BoNT/A1 complex under acidic conditions. **A** Schematic representation of the cryo-EM data processing pipeline. **B** Representative micrograph. Scale bar, 20 nm. **C** Representative 2D class averages. **D** Angular plot of particles used for 3D reconstruction.

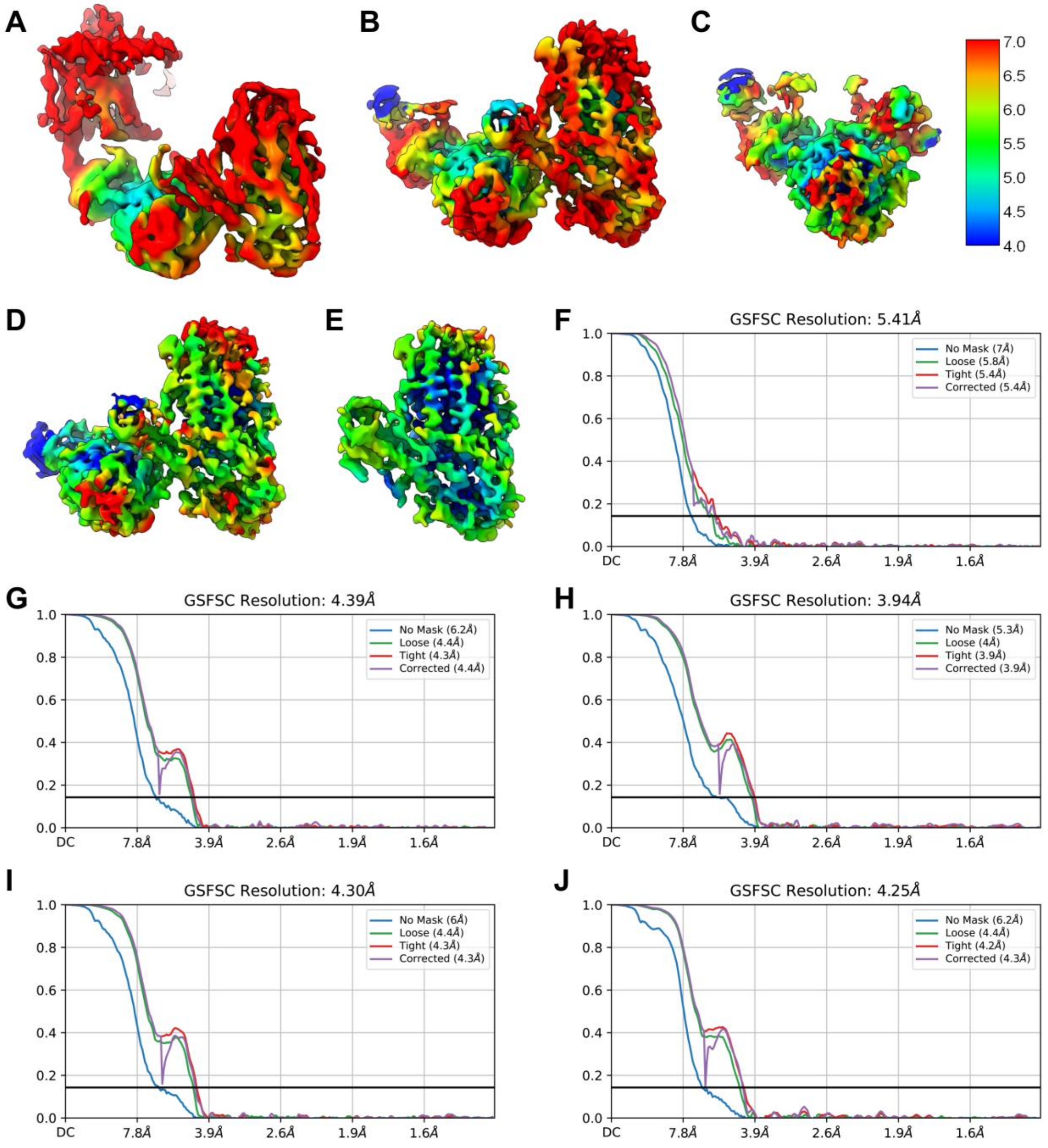

Supplementary Fig. 14. Local resolution and GFSC curve of SV2B-BoNT/A1 complex reconstructions under acidic conditions. **A-E** Local resolution maps of (A) SV2B-BoNT/A1 and after focused refinement of (B) SV2B-LD-BoNT/A1, (C) SV2B-LD-H_CA1, (D) BoNT/A1, and (E) LCH_NA1. **F-J** GFSC curve at 0.143 cut-off for (F) SV2B-BoNT/A1 and after focused refinement of (G) SV2B-LD-BoNT/A1, (H) SV2B-LD-H_CA1, (I) BoNT/A1, and (J) LCH_NA1.

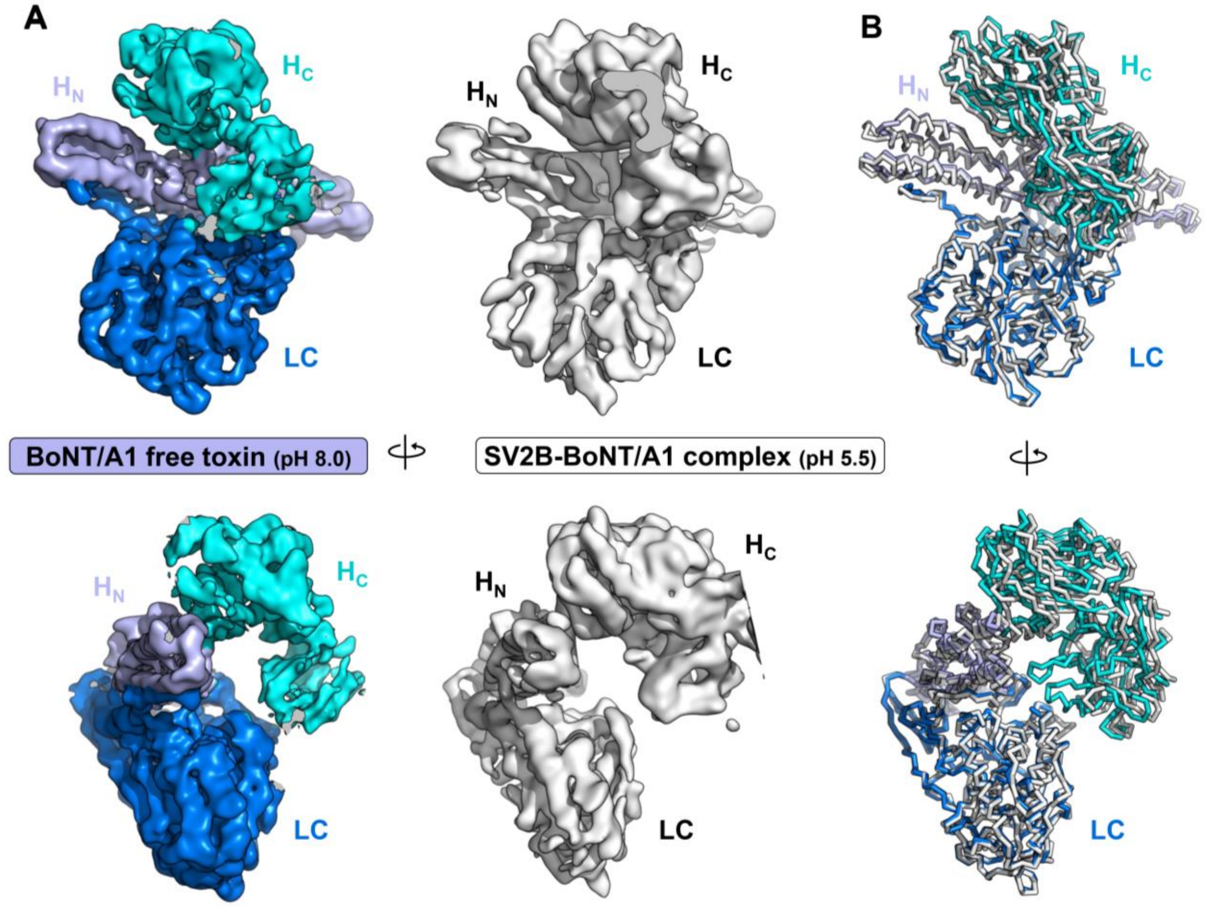

Supplementary Fig. 15. Comparison of semi-closed conformations of BoNT/A1 at pH 8 (free toxin) and pH 5.5 (SV2B-BoNT/A1 complex). **A** Density maps (surface) of BoNT/A1 alone (marine / light blue / cyan) and SV2B-BoNT/A1 (white) at pH 5.5. The maps are contoured at 5σ threshold level. **B** Overlay of BoNT/A1 structural models in free (at pH 8.0) and SV2B-receptor-bound state (at pH 5.5).

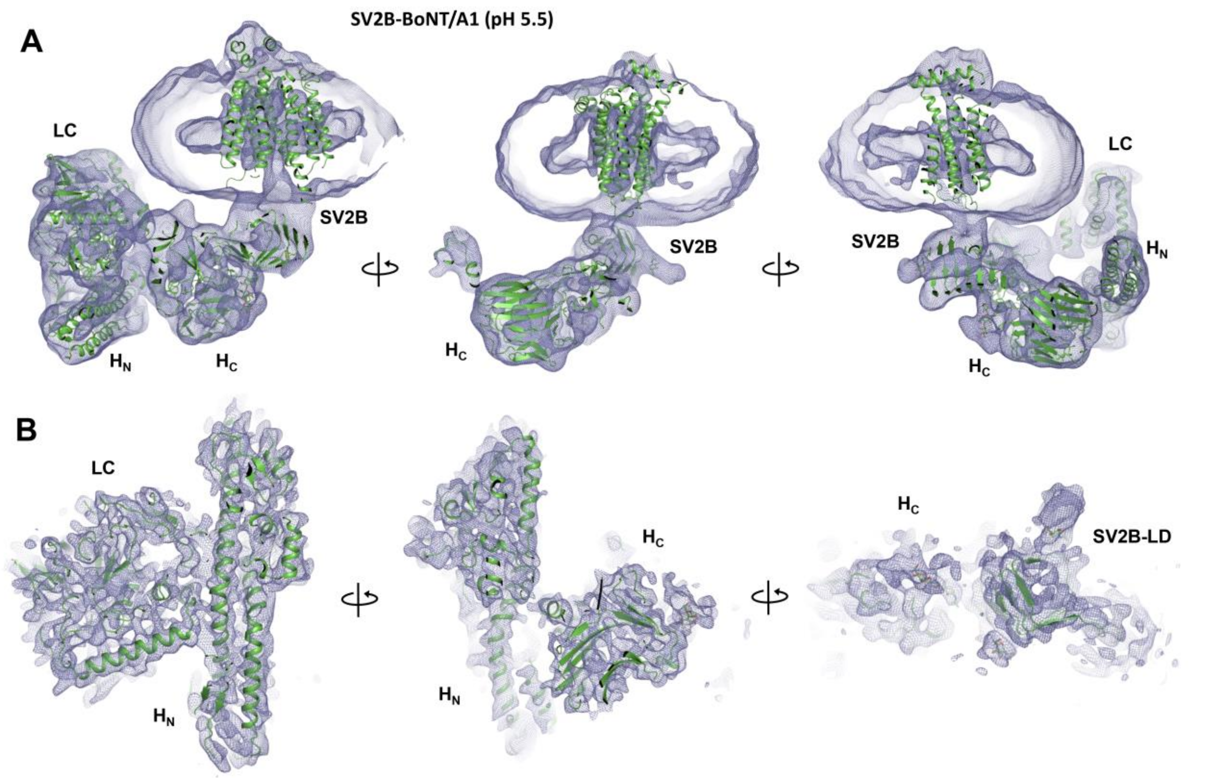

Supplementary Fig. 16. Cryo-EM map features of the SV2B-BoNT/A1 complex at pH 5.5. **A** Density features of the SV2B-BoNT/A1 complex determined at pH 5.5, and **B** after focused refinement. All the maps are contoured at 6σ threshold level.

Supplementary Table 1. Cryo-EM analysis and statistics

Sample	SV2B-H_CA1	BoNT/A1		SV2B-BoNT/A1 (pH 8.0)		SV2B-BoNT/A1 (pH 5.5)				
		Global	Local LCH_NA1	Global	Local SV2B-LD-BoNT/A1	Global	Local SV2B-LD-BoNT/A1	Local SV2B-LD-H_CA1	Local BoNT/A1	Local LCH_NA1
EMDB	EMD-50135	EMD-50139	EMD-50138	EMD-50147	EMD-50146	EMD-50151	EMD-50166	EMD-50158	EMD-50154	EMD-50163
PDB	9F1R	-	9F25	9F2J	9F2B	9F3C	9F2Y			
Data collection and processing										
Magnification	130,000									
Voltage (kV)	300									
Electron Dose (e-/Å2)										
Dataset 1	65	80		58		50				
Dataset 2	58	-		50		50				
Dataset 3	58	-		-		50				
Defocus range (µm)	-0.6 to -2.8									
Pixel size (Å)	0.65									
Map resolution	3.67	3.80	3.70	3.98	3.47	5.41	4.39	3.94	4.30	4.25
FSC threshold	0.143									
Number of particles	185,478	147,560	121176	149,718	129039	187,001				
Map sharpening B-factor (Å)	-121.5	-154.9	-129.9	-85.2	-103.3	-322.2	-181.9	-151.3	-184.3	-222.4
Refinement										
Model composition										
Protein residues	960		830	1800	1359	1766	1336			
Ligands / Others	9		0	9	8	5	5			
Map CC (mask)	0.81		0.77	0.68	0.82	0.61	0.73			
RMS deviation										
Bond length r.m.s.d (Å)	0.003		0.002	0.004	0.004	0.005	0.004			
Bond angle r.m.s.d (°)	0.605		0.526	0.633	0.633	0.788	0.707			
MolProbity score	1.93		1.50	1.89	1.80	2.29	2.09			
Clash score	12.53		9.34	10.88	9.58	16.02	16.42			
Rotamer outlier (%)	0.24		0.13	1.19	0.97	1.66	0.16			
Ramachandran plot										
Favoured (%)	95.37		98.05	95.91	95.76	93.64	94.47			
Allowed (%)	4.63		1.95	4.09	4.24	6.19	5.45			
Outlier (%)	0.00		0	0	0	0.17	0.08			
